# Crohn’s Disease Patients Referred for Home Parenteral Nutrition—A Comprehensive Analysis of 18 Years’ Experience at a National Reference Centre

**DOI:** 10.3390/nu17101697

**Published:** 2025-05-16

**Authors:** Sandra Banasiak, Mariusz Panczyk, Jacek Sobocki, Zuzanna Zaczek

**Affiliations:** 1 Student Research Association for Clinical Nutrition of Medical University of Warsaw, Department of General Surgery and Clinical Nutrition, Centre of Postgraduate Medical Education, 01-813 Warsaw, Poland; 2 Department of Education and Research in Health Sciences, Faculty of Health Sciences, Medical University of Warsaw, 02-091 Warsaw, Poland; mariusz.panczyk@wum.edu.pl; 3 Department of General Surgery and Clinical Nutrition, Centre of Postgraduate Medical Education, 01-813 Warsaw, Poland; 4 Department of Human Nutrition, Faculty of Health Sciences, Medical University of Warsaw, 02-091 Warsaw, Poland

**Keywords:** Crohn’s disease, inflammatory bowel disease, home parenteral nutrition, parenteral nutrition, malnutrition, short bowel syndrome, intestinal failure, prognosis

## Abstract

**Background**: Within 10 years of diagnosis, about 50% of patients with Crohn’s disease (CD) require surgery. Repeated small bowel resections can lead to the development of short bowel syndrome (SBS). It is estimated that 65–75% of CD patients are malnourished. This retrospective observational study was conducted in a Polish reference centre for home parenteral nutrition (HPN). The aim of the study was to investigate the nutritional status and characteristics of patients with CD referred to HPN and to analyse the course of their HPN treatment. **Methods**: The study group consisted of all adult patients (N = 46) with CD who qualified for HPN between November 2004 and April 2022. **Results**: The most common indication for HPN was SBS (n = 27; 58.70%), followed by ineffective gastrointestinal nutrition causing progressive malnutrition (N = 9; 19.57%), fistulas (N = 6; 13.04%), and short bowel syndrome and fistulas (N = 4; 8.70%). According to the results of Subjective Global Assessment (SGA), 47.83% (N = 22) of patients were diagnosed with severe malnutrition, followed by 15 patients (32.61%) with moderate malnutrition. Global Leadership Initiative on Malnutrition (GLIM) criteria showed that 71.73% (n = 33) of patients were malnourished on admission to the HPN centre. All patients received parenteral formulas based on individually tailored prescriptions. The results showed that patients with a stoma received statistically significantly higher PN volumes (*p* = 0.027) and higher amounts of amino acids (*p* = 0.046) and fat emulsion (*p* = 0.046). Septic complications were twice as common as mechanical or metabolic complications, although 43.47% of patients had no complications. At the time of data analysis, 19 patients (41.30%) had been successfully weaned from HPN, of whom 12 (26%) achieved nutritional autonomy after 136–1419 days (mean: 560 ± 380.9). **Conclusions**: Malnutrition is a major problem in CD patients, especially those with SBS. Early nutritional intervention and consideration of artificial nutrition in this study group (HPN) are necessary to prevent the long-term consequences of severe malnutrition. To our knowledge, this was the first study to report on Crohn’s patients referred to long-term HPN. Further studies are needed to assess the impact of HPN on functional, laboratory, and anthropometric outcomes with a view to optimising treatment outcomes.

## 1. Introduction

Crohn’s disease (CD) is one of the two main forms of inflammatory bowel disease (IBD) and is characterised by recurrent inflammation of the gastrointestinal tract, which can occur throughout its entire length but most commonly affects the intestines [1]. According to the Centre for Disease Control and Prevention (CDC), the global prevalence of the disease rose to 6.8 million people in 2017 [2]. Despite the improvement of treatment options for CD symptoms, up to 50% of patients require surgery within 10 years of diagnosis. Indications for small bowel resection include complications such as strictures, fistulas and abscesses as well as treatment resistance. They can all contribute to intestinal failure (IF), including the development of short bowel syndrome (SBS) [3].

SBS is a malabsorptive condition that leads to diarrhoea, dehydration, electrolyte imbalances, nutrient deficiencies and weight loss, eventually leading to the development of malnutrition [3]. Malnutrition affects 65–75% of CD patients, and can occur not only as a result of SBS but also due to the underlying disease itself as a result of reduced intestinal absorption, intestinal dysbiosis, or symptoms such as abdominal pain, loss of appetite, nausea, or vomiting [4]. Inflammation leads to a loss of blood and proteins in the intestinal lumen, and changes in ionic transport lead to a loss of fluids and electrolytes [5]. To meet nutritional requirements and maintain fluid and electrolyte balance in CD patients, particularly those with SBS-IF, long-term total or supplemental parenteral support (PS) is implemented. PN is a life-saving therapy in which nutrients such as amino acids, glucose, lipids, electrolytes, vitamins and trace elements are administered intravenously [6]. The technological advances in the 20th century have made it possible to transfer this method to the home setting as home parenteral nutrition (HPN), which has greatly improved the lives of patients requiring long-term nutritional support, such as those with chronic intestinal failure [7].

There are relatively few data on patients with CD receiving HPN. The purpose of this study was to examine the nutritional status and specific characteristics of patients with Crohn’s disease who require HPN and to analyse the HPN treatment course with regard to selected parameters. The study aims to provide evidence that can guide early nutritional interventions and improve the management of Crohn’s disease patients on HPN.

## 2. Materials and Methods

### 2.1. Study Design and Setting

This retrospective observational study was conducted in a Polish reference centre for HPN and included prospectively collected data from hospitalisation records during HPN qualification and from all medical records of outpatient visits to the HPN centre between November 2004 and April 2022. Patient follow-up extended from the first HPN hospital admission to the last visit on the day of study termination, HPN discontinuation, or death. The study protocol was approved by the Ethics Committee of the Medical University of Warsaw (AKBE/347/2023).

### 2.2. Study Sample

The study group consisted of all consecutive adult patients with CD who qualified for HPN between November 2004 and April 2022. The inclusion criteria required that Crohn’s disease was an initial cause for implementing PN, and patients with cancer as a concomitant disease were excluded from the analysis (N = 3). Based on these criteria, a cohort of 46 patients were included in the analysis.

### 2.3. Institutional Approach to HPN Procedure

The assessment of qualification for home parenteral nutrition took place in the hospital. It included a nutritional status assessment, blood tests, a full physical examination, education and training of the patient and/or carer by a trained nurse, the insertion of a tunnelled central venous catheter (CVC), and prescription and adjustment of the nutritional formula individually prepared by the hospital pharmacy or patient’s carer. The nutritional assessment, including measurements of body weight and height, calculation of body mass loss, and assessment with the SGA scale, was carried out by a trained dietitian within the first 24 h of admission to the ward and repeated every 7–14 days if necessary. After discharge, patients were required to remain in constant contact with the HPN clinic and attend all quarterly follow-up visits.

### 2.4. Data Collection and Nutritional Assessment

The data collected included demographic information such as gender and age at the time of HPN qualification, the date of onset and duration of HPN, and, if applicable, the date and reason for the discontinuation of HPN. Medical data, such as concomitant diseases, the indications for parenteral nutrition, and the anatomical details of the bowel, including the presence or absence of fistulas, stomas, and the type of SBS, were also collected. If the patient’s bowel anatomy changed during the study, both the initial and final parameters were recorded. The laboratory data collected included serum albumin, total protein, C-reactive protein, leukocytes, lymphocytes, and haematocrit. Anthropometric measurements such as weight and height as well as nutritional assessment data including unintentional weight loss (kg and % of body mass) were also analysed. For patients who qualified for the HPN procedure since 2012—the start of mandatory use of the Subjective Global Assessment (SGA) to assess nutritional status—scale results were collected. For patients who qualified prior to 2012, the SGA was performed by a trained clinical dietitian during data analysis based on the medical records obtained.

Body mass index (BMI) was calculated by dividing weight (kg) by height (m) squared [7]. BMI categories were classified according to CDC criteria: underweight (BMI < 18.5 kg/m^2^), normal weight (BMI ≥ 18.5 kg/m^2^–24.9 kg/m^2^), or overweight (BMI ≥ 25.0 kg/m^2^). The SGA results were interpreted as follows: A—well-nourished; B—moderately malnourished (or suspected of being malnourished); C—severely malnourished; D—high risk of malnutrition. Inflammation was detected when the C-reactive protein (CRP) level exceeded 10 mg/L, and loss of muscle mass was assessed based on physical examination data via the SGA scale and deterioration of the patient’s physical condition.

Based on the serum albumin level, body weight, and ideal body weight (calculated according to the Lorentz formula [8]), the Nutritional Risk Index (NRI) in patients under 65 years of age and the Geriatric Nutritional Risk Index (GNRI) in patients over 65 years of age were calculated according to the following formulas:

NRI = (1.519 × serum albumin) (g/L) + 41.7 × (present weight/ideal body weight)

GNRI = (1.489 × serum albumin) (g/L) + 41.7 × (present weight/ideal weight)

Patients with an NRI score >100 were considered to be at no nutritional risk, 97.5–100 at mild risk, 83.5–97.5 at moderate risk, and <83.5 at major nutritional risk [9]. Patients with a GNRI score >98 were classified as at no risk, 92 to ≤ 98 as at low risk, 82 to <92 as at moderate risk, and <82 as at major risk [10].

Total lymphocyte count (TLC) was determined according to the following formula:

TLC = (% lymphocytes × leukocytes)/100

The cut-off values for total lymphocyte count used for the classification of immunological depletion were as follows: <800 cells/mm^3^—severe depletion, 800–1999 cells/mm^3^—moderate depletion, and >2000 cells/mm^3^—no immunological depletion [11].

Also, the Prognostic Nutritional Index was calculated according the following formula:

PNI = 10 × serum albumin (g/dL) + 0.005 × total lymphocyte count (mL)

The cutoff points for PNI were as follows: PNI <35—severe risk, PNI <38—moderate risk, PNI >38—normal risk.

### 2.5. Stastical Analysis

In this study, descriptive statistics were employed to succinctly summarise the characteristics and measurements of the study population. For categorical variables such as gender or specific medical indications, the analysis included the total number of observations (N) and the percentage (%). For continuous variables like body mass index (BMI) and serum levels, the mean (M) and standard deviation (SD) were calculated to describe the central tendency and dispersion, respectively. Additionally, the median (Mdn) and range (Min–Max) were determined to highlight the central value and the overall spread of data. The semi-interquartile range (IQR/2) provided a robust measure of variability.

Pearson’s chi-squared test with a continuity correction and the two-sided Fisher’s exact test were strategically employed to analyse associations among categorical variables, such as gender differences within the study groups and specific indications for home parenteral nutrition (HPN). Pearson’s test is crucial for detecting relationships and assessing the independence of these variables. For contingency tables with small sample sizes or uneven distribution among cells, Fisher’s exact test provided a more precise *p*-value.

For continuous data that did not meet normal distribution assumptions, the Brunner–Munzel test was applied. Utilizing random permutations, this test is essential for comparing differences between two independent samples and especially useful in the analysis of anthropometric measurements.

To investigate correlations, Spearman’s rank correlation coefficient was used to estimate the relationship between age at study enrolment and variables such as BMI and body mass. This non-parametric measure is particularly effective for data that do not exhibit linear relationships or a normal distribution, providing a reliable assessment of monotonic relationships.

For differences across multiple groups, the Kruskal–Wallis rank test was utilised. This non-parametric test is suited for assessing statistical differences across samples that do not adhere to a normal distribution and was employed to analyse variations based on factors such as the type of short bowel syndrome (SBS), the presence of a stoma, and other clinical parameters relevant to HPN. For the SBS-JC group, which consisted of only two patients, the median was calculated as the mean of the two data points, and the interquartile range (IQR) was derived by treating the two values as the first (Q1) and third quartiles (Q3). Due to the small sample size, these values should be interpreted with caution.

In all analyses conducted within this study, a significance level of 0.05 was maintained, meaning that results were considered statistically significant if the probability of occurrence by chance was less than 5%. Additionally, the statistical analyses were performed using Jamovi (Version 2.5) [12].

## 3. Results

### 3.1. Characteristics of the Study Group

A total of 46 patients met the inclusion criteria for the study. The gender distribution was symmetrical. The age of the patients ranged from 21 to 79 years, with a mean of 39.67 ± 13.98. There were no statistically significant differences between the genders in terms of age at the time of qualification (*p* = 0.757).

Short bowel syndrome was the most common indication for HPN (N = 27; 58.70%), and other causes were as follows: ineffective gastrointestinal nutrition causing progressive malnutrition, 19.57% (N = 9); fistulas, 13.04% (N = 6); and short bowel syndrome and fistulas, 8.70% (N = 4). No significant differences were found in the indications for HPN, the types of SBS, or the presence of a stoma according to the age of the participants. The detailed characteristics of the study group are shown in Table 1.

### 3.2. Anthropometric Measurements and Nutritional Assessment

The mean body mass at admission ranged from 32 to 120 kg, with a mean of 54.98 ± 15.89 and no statistically significant differences according to gender. According to BMI classification, 50% (N = 23) of patients were undernourished. In 15 patients (32.61%, 9 females and 6 males), BMI was lower than 17 kg/m^2^. In particular, six patients (13.04%) had a BMI below 15 kg/m^2^ and two patients (4.35%) had one below 12 kg/m^2^. The median BMI was 18.35 ± 2.12 kg/m^2^. A positive correlation with age was found for both BMI (*p* = 0.022) and body mass (*p* = 0.033), indicating that, in our study population, older Crohn’s disease patients tend to have a higher BMI and body mass compared to younger patients. This may suggest that, as patients age, survivors of the disease may have relatively better nutritional status, or that age-related factors contribute to higher body mass and BMI. The anthropometric measurements are summarised in Table 2 and Table 3.

The correlation between anthropometric measurements and indication for HPN, type of SBS, and presence of stoma was also evaluated, and no significant differences were observed. However, the lack of any loss of body mass prior to HPN initiation was observed only in patients with jejunoileal anastomosis with intact colon.

Assessment of nutritional status using the GLIM criteria [13] showed that 71.73% (n = 33) of patients were malnourished on admission to hospital for qualification for the HPN procedure. According to SGA, the majority of patients were diagnosed with severe malnutrition (N = 22, 47.83%), followed by 15 patients (32.61%) with moderate malnutrition (or suspected malnutrition), 6 patients (13.04%) with high risk of malnutrition and 3 patients (6.52%) with proper nutritional status. No significant differences were found in SGA results between genders (χ^2^ = *p* = 0.901) and in relation to age (*p* = 0.836; Kruskal–Wallis rank test), indication for HPN (χ^2^ = 1.148, *p* = 0.765), type of SBS (χ^2^ =1.045, *p* = 0.903), as well as presence of stoma (χ^2^ =3.450, *p* = 0.063). The distribution of SGA categories with regard to indication for HPN and SBS type is shown in Table 4.

Among the variables within the SGA, swelling was more common in women (N = 10; 43.48%) than in men (N = 1; 4.35%) and the difference was statistically significant (χ^2^ *p* = 0.002). In addition to the above measures, total lymphocyte count and indicators of nutritional risk, such as PNI, NRI, and GNRI, were calculated. The results are shown in Table 5, Table 6, Table 7 and Table 8.

### 3.3. Laboratory Tests

According to the analysis of blood tests carried out before the start of nutritional therapy, inflammation occurred in 65.20% of patients on admission to the HPN centre (N = 30). Serum albumin levels were below the normal range in 35 patients and below 2.4 g/dL in 19. There were no statistically significant differences in either result according to gender, age, type of short bowel syndrome, or the presence of a stoma. However, the total protein values differed significantly according to the indication for HPN (*p* = 0.006). The results of the laboratory tests are summarised in Table 9, Table 10, Table 11, Table 12 and Table 13.

### 3.4. Parenteral and Oral Nutrition

All patients received home parenteral nutrition via a tunnelled CVC that was inserted during hospitalisation. Apart from parenteral nutrition, 13 (28.26%) people could eat orally without restrictions, 8 (17.40%) with restrictions, and the rest (N = 25, 54.34%) nil per os. There were no significant differences in the composition of the parenteral formulae according to age and gender. Details of the composition of the parenteral formulae, including daily volume, osmolarity, energy and amino acid content, non-protein energy, and fat emulsion volume, are shown in Table 14, Table 15 and Table 16. The composition of the parenteral admixture of one patient was not available in the medical documentation.

The majority of patients (N = 29, 64.44%) were prescribed SMOF fat emulsion, nine patients (20%) received a fat emulsion based on soybean oil, six patients (13.33%) received one based on olive oil, and one patient (2.22%) received a formula with an emulsion containing only fish oil. There were no statistically significant differences between the genders in the type of emulsion (χ^2^ = 4.735, *p* = 0.192).

The composition of the parenteral formulas was analysed with regard to intestinal anatomy and SBS type. The results showed that patients with a stoma received statistically significantly higher PN volumes as well as a higher content of amino acids and fat emulsion (Table 17).

Considering the type of SBS, the results showed that patients with terminal jejunostomy and ileostomy received higher PN volumes as well as higher total and non-protein energy content and amino acid content, but the differences were not statistically significant, as shown in Table 18.

The analysis of the nutritional admixtures’ compositions in relation to the nutritional status according to the SGA results on admission showed no significant differences.

During the course of HPN, 50% of patients (N = 23) required no changes in PN volume, 45.65% (N = 21) required no changes in energy content, 69.57% (N = 32) required no changes in amino acid content, and 73.91% (N = 34) required no changes in the amount of fat emulsion. An increase in energy content was required in 14 patients (30.43%), 7 patients (15.22%) were prescribed a higher amount of amino acids, and 8 patients (17.39%) were prescribed a higher amount of fat emulsion. The changes in prescriptions did not differ statistically by gender or age.

The mean duration of home parenteral nutrition in the study group was 1759 ± 1754.29 days, with a minimum duration of 136 days and a maximum duration of 6857 days when the data were analysed. The median duration of HPN treatment was 645.0 ± 1293.0 days in women and 2434.0 ± 1527.0 days in men, and the difference was statistically significant (Brunner–Munzel test: 2.179, *p* = 0.036). No significant differences were found with regard to the age of the patients or the formation of the stoma. The median duration of HPN was longer in patients referred to the procedure due to SBS and SBS with coexisting fistulae than in patients with fistulae and inefficient enteral feeding (1527 ± 2424.5 days and 1459.5 ± 1758.5 days versus 854.5 ± 192.25 days and 565 ± 527 days, respectively), but the difference was not statistically significant. In terms of bowel anatomy and type of SBS, the median HPN duration was longest in patients with SBS-JC (2878.0 ± 768.0 days) and SBS-JIC (2514.0 ± 2257.0 days) and shortest in patients with ileostomy (613.5 ± 814.8 days) and in patients without SBS (851.0 ± 2661.0 days), but this difference was also not statistically significant.

The average number of hospitalisations was 2.5 (±3.20). A total of 14 patients (30.83%) did not require hospitalisation during the course of HPN, 9 patients (19.57%) were hospitalised once, 8 patients (17.39%) twice, and 5 patients (10.87%) three times. The highest number of hospitalisations was 11 and 15, each required by one patient (2.17%). The median number of hospitalisations was four times higher in men (4.0 ± 2.0) than in women (1.0 ± 2.0) (*p* = 0.043). No statistical differences in the number of hospitalisations were found in relation to the type of SBS, the presence or absence of a stoma, or the indications for HPN.

Septic complications (N = 20) were twice as common as mechanical (N = 7) and metabolic (N = 2) complications. A total of 20 patients (43.47%) had no complications during HPN, 14 patients (30.43%) had a complication once, and 5 patients (10.86%) had a complication twice. Septic complications included catheter-related bloodstream infections (CRBSI) and catheter tunnel phlegmon, mechanical complications included, e.g., catheter protrusion or prolapse, and metabolic complications included water–electrolyte and metabolic disturbances. There were no statistically significant differences in the number or type of complications according to gender, age, type of SBS, presence or absence of a stoma, or reason for inclusion in HPN. The type and number of complications as a function of gender are shown in Table 19.

At the time of data analysis, 21 patients (45.65%) were still receiving HPN, 19 patients (41.30%) were weaned from parenteral support, 2 patients (4.08%) died, and 1 patient was lost to follow-up. The most common reason for weaning from PN was the achievement of gastrointestinal autonomy (N = 12), and five patients ended HPN treatment at their own request. The reason for treatment cessation was not statistically related to age (*p* = 0.300) or patient gender (*p* = 0.764). The duration of HPN treatment with regard to the cause of treatment discontinuation is shown in Table 20.

## 4. Discussion

This study included 46 patients with Crohn’s disease who qualified for home parenteral nutrition at a reference centre in Poland over a period of 18 years. There are relatively few data on the nutritional status of CD patients referred to long-term PN. In an observational study by Kurin et al. [14], the authors reported that 25 of 2359 (1.1%) patients with IBD received long-term HPN between 2009 and 2015, and the group included 24 patients with CD (96%). Considering the duration of the analysis, the number appears to be higher than in this study (4 vs. 2.5 patients per year). In the study conducted in Minnesota by Bakhshi et al. [15], less than 4% of the studied CD patients required HPN, and among them, 78.6% had moderate-to-severe CD. In a 2016 analysis by Brandt et al. [16], CD patients accounted for 24% (N = 121) of all patients receiving HPN between 1970 and 2010 (approximately three patients per year). These results suggest that, while IBD patients requiring HPN represent only a small proportion of the total IBD population, IBD and Crohn’s disease in particular represent a significant proportion of the total HPN population. This is supported by the data from Pironi et al. [17], who reported SBS as the most common pathophysiological mechanism of IF and Crohn’s disease as the most common underlying disease (22.4%). Accordingly, SBS was the main reason (58.7% of cases) for qualifying for HPN in this study, with SBS-I (30.43%) and SBS-J (17.39%) dominating. This is consistent with the results of the Pironi study mentioned above, where SBS-J was present in 38.6% of cases. In our experience, many hospitals referring patients for HPN do not provide sufficient information on the length of the remaining bowel and refer to the end jejunostomy as an end ileostomy. Therefore, we assume that at least some of the cases we reported as SBS-I (according to the referring hospitals’ medical documentation) were in fact SBS-J.

The assessment of nutritional status based on the SGA results revealed that 60.87% of patients were malnourished and almost half of the patients (47.83%) were severely malnourished on admission to the HPN unit. These results are worrying, especially as malnutrition is known to be associated with a poorer prognosis and higher mortality and complication rates, as well as a higher number of hospitalisations and a deterioration in quality of life [18]. In comparison, Liu et al. [19] reported malnutrition in 49.5% of IBD patients, but the study was conducted in an Asian population, so phenotypic differences must be taken into account. The results of Casanova et al. [20] showed that of 333 patients with IBD, 57% of whom had CD, only 7% received SGA grade B or C and the overall prevalence of malnutrition was 16% [95% CI = 12–20%], with no significant differences between CD and UC. In the recent study by Viganò et al. [21], the authors observed disease-related malnutrition in 26.3% of CD patients. However, the patients in both studies mentioned did not receive home parenteral nutrition, suggesting that CD patients referred for HPN are in a much more severe condition. It should also be borne in mind that SGA is based on a subjective view of the interviewer, which is another possible explanation for the differences between different studies. Therefore, we believe that double-checking with other assessment methods is of clinical importance to ensure the objectivity of the results.

In the present study, the median BMI was slightly below normal values (18.35 ± 2.12 kg/m^2^). Undernutrition according to BMI values was found in 50% of patients, and 32.61% of patients had a BMI of less than 17 kg/m^2^, indicating severe malnutrition. In addition, six patients (13.04%) had BMI values below 15 kg/m^2^ and two patients (4.35%) had BMI values below 12 kg/m^2^, indicating severe malnutrition. In the study by Pironi et al. [17], 15.1% of HPN patients with CIF were found to have a BMI between 15.1 and 18.5 kg/m^2^, while a BMI < 15 kg/m^2^ was found in only 2.4% of patients, but the authors did not provide a separate analysis of the distribution of BMI categories in CD patients receiving HPN. The results of the study by Xu et al. [22] found a BMI < 18.5 kg/m^2^ in 44.6% of patients with perianal fistulising CD, while Papadimitriou et. al. [23] reported a median BMI of 25.00 kg/m^2^ (23.30–29.40) in the group of 53 CD patients, of whom 79.1% were in remission, 14.6% had mild disease severity, and 6.3% had moderate. These differences support the hypothesis that the incidence of malnutrition in Crohn’s disease patients varies according to overall condition and disease severity, presenting HPN-dependent patients as “the sickest of the sick”. This is of particular concern as a low BMI (<17.0 kg/m^2^) in CD patients is associated with an increased risk of postoperative infectious complications, as reported by Zhu et al. [24]. In addition, patients with higher BMI values were found to have a lower rate of penetrating disease behaviour [25]. Although BMI is one of the most commonly used nutritional indicators, it only takes into account the weight and height of patients and, if used alone, can lead to false-negative results in terms of the diagnosis, especially in patients with swelling. For this reason, we also included other indicators of nutritional status in this study. The analysis of another nutritional measure, Prognostic Nutritional Index, proved that the patients in the study group were at severe nutritional risk, as the mean PNI was 30.53 ± 11.03 and 63% of patients (N = 29) had a PNI below 35. Low PNI was widely reported as a poor prognostic factor in cancer patients [26], but its use in CD is rarely discussed. The results provided by Duan et al. [27] presented PNI as a useful indicator for predicting surgical recurrence in CD patients, but not as an indicator for predicting postoperative complications. Nonetheless, according to the data from Zhou et al. [28], the incidence of postoperative overall and infectious complications was higher in patients with PNI < 40 than in those with PNI ≥ 40 (50.0% and 46.7% vs. 23.3% and 16.3%; *p* = 0.018 and *p* = 0.005, respectively). These data provide a further rationale for earlier nutritional assessment and intervention in CD patients [19,29]. Although the data on the use of PNI to predict complications in CD patients are inconclusive and emphasise the need for further studies, we believe that it is a valuable indicator of nutritional status that takes into account not only anthropometric variables but also reflects immunological aspects, and that its introduction into the clinical practise of nutritional risk assessment would be beneficial.

Analysis of the blood tests revealed that the majority of patients (65.20%) had inflammation (CRP > 10 g/L) on admission to hospital. This is understandable and to be expected given the mechanism of the disease itself and the fact that patients underwent bowel resection or developed fistulae. It is very likely that the presence of inflammation also contributed to the development of malnutrition in the study group, as it stimulates catabolism. The mean serum albumin level was 29.2 ± 1.09 g/L, and levels below 35 g/L were found in 71.74% of patients. This is consistent with the findings of Bakhshi et al. [15], who reported a mean albumin level of 3.3 g/dL (95% CI: 3–3.5). Su et al. [30] found significantly lower levels of serum albumin and CRP in CD patients compared to healthy controls. In addition, the authors reported a negative correlation between serum albumin levels and disease activity in CD patients and a positive correlation between serum CRP levels and disease activity in CD patients. Both CRP ≥ 10 mg/L and serum albumin < 33.6 g/L were found to be independent risk factors for postoperative infectious complications in the aforementioned study by Zhu et al. [24]. The authors concluded that normalising serum albumin and CRP levels could reduce the incidence of postoperative infectious complications. Although the use of serum albumin alone as an indicator of nutritional status is not recommended, it is of value as part of the NRI, GNRI, and PNI indicators.

The composition of the parenteral admixtures was also analysed in this study. The mean daily PN volume and energy content were 2454.56 ± 469.46 mL and 1381.00 ± 203.09 kcal, respectively, and the mean energy requirement per kg body weight was 26.71± 7.42 kcal/kg bw per day. In comparison, the values in the study by Pironi et al. [17] were 1877 ± 1016 mL/day and 1088 ± 649 kcal/day, respectively, and the patients received an average of 18.7 ± 11.9 kcal/kg bw/day. It should be remembered that the study group in Pironi’s work consisted of different patients receiving HPN due to CIF and not only CD patients. According to the ESPEN recommendations for clinical nutrition in IBD (recommendation 6, recommendation grade GPP—strong consensus (95% agreement)), energy intake should be 30–35 kcal/kg bw/day, which was not the case in the two studies compared. In contrast to Pironi et al., who observed a decrease in PN volume with increasing age category and differences in PN volume according to the pathophysiological mechanism, we reported no statistically significant differences in the composition of the PN formula according to age and indication for HPN. Nevertheless, we observed a similar trend towards higher PN volumes and energy content in patients with SBS and coexisting fistulas, and higher volume, energy, and amino acid content in patients with SBS-J. It should be noted that the methodology and statistical methods used differed between those studies.

In this study, we report a median duration of HPN of 1759 ± 1754.29 days. The first patient with CD qualified for HPN in 2004 and was still receiving parenteral support at the time of data analysis. Weaning from parenteral support was possible after 560 ± 380.9 days of HPN in 41.30% of patients, mostly (63.16%) due to the achievement of gastrointestinal autonomy. Restorative surgery was performed in seven patients (15%), corresponding to 58.33% of patients who regained intestinal autonomy. In comparison, 24% of IBD patients were weaned from HPN in the Danish study by Brandt et al. [16], and 8% of IBD patients regained bowel autonomy after 124 days of HPN treatment following restorative surgery, while spontaneous adaptation was the cause of weaning from HPN at 318 days after the last bowel resection in 19% of IBD patients. The authors reported that 29% of IBD patients died during HPN treatment of approximately 1811 days over an observation period of 40 years, while in our study, 13.04% of patients died over a period of 18 years after receiving HPN for 535 ± 77.1 days. In the study by Watanabe et al. [31], the mortality rate was reported to be 9% of patients over a period of 19 years after a median of 14.9 HPN years. It should be noted that the authors provided data on causes of death, whereas we were not able to do so, as the patients’ relatives are not obliged to provide such information to the HPN centre, so our data on this topic are limited. In addition, the data from the Danish study refers to IBD patients with both CD and ulcerative colitis, whereas our study only included CD patients. In the study by Watanabe et al., 57% of patients with CD who were weaned off HPN achieved this after at least 2 years (730 days) of HPN treatment.

Over an observation period of 18 years, we reported that HPN-related complications occurred in 56.53% of patients, with septic complications reported in 43.47% of patients. These figures are lower than in the study by Bakhshi et al. [15], who reported a total of 71.4% of patients with CRBSI over a 31-year observation period. The authors also reported an average of six hospitalisations, which is more than twice as high as in our study. In addition, no cases of CVC-related thrombosis or parenteral nutrition-associated liver disease (PNALD) were observed in this study, whereas Bakhshi et al. reported these in 21.4% and 21% of patients, respectively. Similar data came from a study by Watanabe et al. [13], who analysed the outcomes of HPN in CD patients over 19 years. They reported that CRBSI occurred in 61% of the 21 patients who made up the study group and had an incidence of 0.32/1000 catheter days. More than half of the patients received PN via a central venous port device, whereas in our study, all patients had a tunnelled CVC implemented on initial hospitalisation. The authors reported liver disease as the second most common complication, occurring in 38% of patients. The difference in the incidence of complications between the studies may be related to many factors, such as the type of intravenous lines used, the type of parenteral nutrition admixtures administered (ready-to-use vs. individually prescribed formulas), their composition, and differences in the training of carers and the institutional approach to HPN, which were not described in the aforementioned papers. We believe that the significantly lower incidence of liver complications compared to other studies is due to the better tailored dosing of fat emulsions (median: 100 ± 25 mL of a 20% emulsion).

The strengths of our study include its duration and a well-described cohort of CD patients receiving HPN from a specific geographic area that is likely representative of the general population. The treatment of the patients was standardised as the work originated from one centre (there are few HPN centres with such a scope). The study was comprehensive and considered multiple clinical parameters. Nevertheless, our study had some limitations, such as its retrospective nature, which meant that we did not have access to detailed medical records from other medical institutions, including the CD treatments used and detailed information regarding the patients’ oral diet.

The sample size is relatively small; however, no better evidence exists. Therefore, the results should be interpreted with caution. In particular, regarding patients with SBS-JIC, the median and interquartile range (IQR) were calculated from only two data points, which is unconventional and offers limited statistical insight.

## 5. Conclusions

This is one of the few studies reporting on the nutritional status of patients with Crohn’s disease referred to HPN. The results show that a subgroup of CD patients requiring home parenteral nutrition is characterised by poor nutritional status parameters. These results strongly emphasise the importance of regular screening for malnutrition using validated tools such as SGA or GLIM criteria as well as indicators such as NRI, GNRI, and PNI. These tools assess various parameters, including weight loss, body mass index, laboratory results, and food intake, to identify patients at risk of malnutrition. Such screening should be included in the routine care of patients with Crohn’s disease to prevent the development of malnutrition and its consequences and to ensure the early initiation of nutritional therapy as soon as malnutrition or the risk of malnutrition is detected. Nutritional therapy may include dietary modification, oral nutritional supplements, or enteral nutrition. In cases where these methods are ineffective or patients are unable to meet their nutritional requirements, HPN should be considered as a last resort, particularly in patients with intestinal failure. However, further, preferably prospective, studies that include other methods of measuring nutritional status and body composition are needed.

## Figures and Tables

**Table 1:** Clinical characteristics of the study group, including indication for home parenteral nutrition (HPN), short bowel syndrome (SBS) type, and presence of stoma, compared between females and males.

	Female		Male		χ^2^	*p*-Value *
	N	%	N	%		
Indication for HPN						
SBS	14	60.87	13	56.52	1.148	0.765
Fistulas	3	13.04	3	13.04		
Inefficient enteral nutrition	5	21.74	4	17.39		
SBS and fistulas	1	4.35	3	13.04		
SBS type **						
SBS-J	3	34.78	5	39.13	1.045	0.903
SBS-JC	1	13.04	1	21.74		
SBS-JIC	3	4.35	2	4.35		
SBS-I	8	13.04	6	8.70		
Presence of stoma						
no	18	78.26	12	52.17	3.450	0.063
yes	5	21.74	11	47.83		

* Pearson’s chi-squared test with a continuity correction; ** SBS-J (short bowel syndrome with end jejunostomy), SBS-JC (short bowel syndrome with jejunocolic anastomosis), SBS-JIC (short bowel syndrome with jejunoileal anastomosis with an intact colon), SBS-I (short bowel syndrome with end ileostomy).

**Table 2:** Anthropometric variables according to gender.

Anthropometric Variable	Female		Male		Statistic	*p*-Value *
	Mdn	IQR	Mdn	IQR		
Height (cm)	165.0	10.0	176.0	10.5	5.840	<0.001
Body mass (kg)	50.0	15.3	57.0	13.0	1.161	0.260
BMI (kg/m^2^)	19.1	4.4	17.6	2.62	−0.539	0.578
Loss of body mass (kg)	8.0	6.0	15.0	6.0	1.981	0.057
Loss of body mass (%)	15.2	11.0	17.0	10.0	1.296	0.213

Mdn—median, IQR—interquartile range; * Brunner–Munzel test.

**Table 3:** Correlation between anthropometric variables and age.

Anthropometric Variable	Spearman’s Rho	Degrees of Freedom	*p*-Value *
Height (cm)	−0.076	44	0.615
Body mass (kg)	0.314	44	0.033
BMI (kg/m^2^)	0.337	44	0.022
Loss of body mass (kg)	0.115	38	0.479
Loss of body mass (%)	−0.023	38	0.889

* Spearman’s rank correlation coefficient.

**Table 4:** The distribution of SGA categories with regard to indication for HPN and SBS type.

	SGA									
	A		B		C		D		χ^2^	*p*-Value *
	N	%	N	%	N	%	N	%		
Indication for HPN										
SBS	3	11.11	10	37.04	4	14.81	10	37.04	7.267	0.609
Fistulas	0	0.00	2	33.33	1	16.67	3	50.00		
Inefficient enteral nutrition	0	0.00	3	33.33	0	0.00	6	66.67		
SBS + fistulas	0	0.00	0	0.00	1	25.00	3	75.00		
SBS type **										
No SBS	0	0.00	6	35.29	3	17.65	8	47.06	9.077	0.696
SBS-J	1	12.50	2	25.00	0	0.00	5	62.50		
SBS-JC	0	0.00	1	50.00	1	50.00	0	0.00		
SBS-JIC	1	20.00	2	40.00	0	0.00	2	40.00		
SBS-I	1	7.14	4	28.57	2	14.29	7	50.00		

* Pearson’s chi-squared test with a continuity correction; ** SBS-J (short bowel syndrome with end jejunostomy), SBS-JC (short bowel syndrome with jejunocolic anastomosis), SBS-JIC (short bowel syndrome with jejunoileal anastomosis with an intact colon), SBS-I (short bowel syndrome with end ileostomy).

**Table 5:** Summary of TLC, NRI/GNRI, and PNI results.

	N	M	SD	Mdn	IQR/2	Min	Max
NRI/GNRI	46	80.88	18.86	79.51	13.03	53.90	149.07
PNI	46	30.53	11.03	30.48	6.46	12.33	78.40
TLC	46	2674.92	4930.05	1591.28	2724.16	724.16	33,603.00

M—mean, SD—standard deviation, Mdn—median, IQR/2—semi-interquartile range, Min–Max—range, NRI—Nutritional Risk Index, GNRI—Geriatric Nutritional Risk Index, PNI—Prognostic Nutritional Index, TLC—Total Leukocyte Count.

**Table 6:** The results of NRI/GNRI, PNI, and TLC according to gender.

	Female		Male		Statistic	*p*-Value *
	Mdn	IQR	Mdn	IQR		
NRI/GNRI	80.70	29.40	78.40	21.0	−0.530	0.590
PNI	26.30	13.10	31.10	11.5	0.376	0.691
TLC	17.0	1387.0	1520.0	1346.0	−0.837	0.415

Mdn—median, IQR—interquartile range, NRI—Nutritional Risk Index, GNRI—Geriatric Nutritional Risk Index, PNI—Prognostic Nutritional Index, TLC—Total Leukocyte Count; * Brunner–Munzel test.

**Table 7:** The results of NRI/GNRI, PNI, and TLC according to indication for HPN and SBS type.

Variable		Mdn	IQR	Stat.	*p*-Value *
	Indication for HPN				
PNI	SBS	33.20	14.27	3.360	0.339
	Fistulas	29.20	10.15		
	Inefficient enteral nutrition	24.20	10.71		
	SBS and fistulas	32.80	9.43		
NRI/GNRI	SBS	85.00	24.06	6.370	0.095
	Fistulas	68.90	18.02		
	Inefficient enteral nutrition	66.90	17.14		
	SBS and fistulas	79.50	18.72		
TLC	SBS	1487.30	1167.32	5.590	0.133
	Fistulas	4453.50	1796.24		
	Inefficient enteral nutrition	1345.80	1239.02		
	SBS and fistulas	1547.40	1191.51		
	Type of SBS **				
NRI/GNRI	No SBS	77.82	26.72	6.040	0.196
	SBS-J	73.22	19.28		
	SBS-JC ***	84.42	28.69		
	SBS-JIC	86.17	22.25		
	SBS-I	85.19	18.86		
PNI	No SBS	31.07	10.37	6.500	0.165
	SBS-J	27.93	5.60		
	SBS-JC ***	17.06	2.88		
	SBS-JIC	36.01	18.01		
	SBS-I	34.63	13.67		
TLC	No SBS	1801.22	3340.00	3.580	0.466
	SBS-J	2056.32	1336.61		
	SBS-JC ***	1116.93	767.14		
	SBS-JIC	1487.25	2020.41		
	SBS-I	1295.98	929.45		

Mdn—median, IQR—interquartile range, NRI—Nutritional Risk Index, GNRI—Geriatric Nutritional Risk Index, PNI—Prognostic Nutritional Index, TLC—Total Leukocyte Count; * Kruskal–Wallis rank test; ** SBS-J (short bowel syndrome with end jejunostomy), SBS-JC (short bowel syndrome with jejunocolic anastomosis), SBS-JIC (short bowel syndrome with jejunoileal anastomosis with an intact colon), SBS-I (short bowel syndrome with end ileostomy); *** SBS-JC values are not based on the median but represent the mean of two data points. Similarly, the interquartile range (IQR) for SBS-JC was calculated using these two values as the first (Q1) and third quartiles (Q3). Given the limited sample size, these values should be interpreted with caution.

**Table 8:** The results of NRI/GNRI, PNI, and TLC according to the presence of a stoma.

	Lack of Stoma		Presence of Stoma		Statistic	*p*-Value *
	Mdn	IQR	Mdn	IQR		
NRI/GNRI	78.2	26.4	82.8	21.6	−0.204	0.842
PNI	30.5	15.0	30.4	11.4	−0.142	0.883
TLC	1843.0	1606.0	1211.0	870.0	−1.303	0.203

Mdn—median, IQR—interquartile range, NRI—Nutritional Risk Index, GNRI—Geriatric Nutritional Risk Index, PNI—Prognostic Nutritional Index, TLC—Total Leukocyte Count; * Brunner–Munzel test.

**Table 9:** Characteristics of the study group including albumin level, C-reactive protein (CRP), total protein, and haematocrit.

	N	M	SD	Mdn	IQR/2	Min	Max
Serum albumin (g/dL)	46	2.92	1.09	2.90	0.65	1.20	7.60
CRP (mg/L)	46	50.19	65.19	19.67	35.73	0.67	276.75
Total protein (g/L)	46	7.47	3.72	7.00	0.65	4.40	31.00
Haematocrit (%)	46	34.47	5.35	33.70	3.15	24.70	48.80

M—mean, SD—standard deviation, Mdn—median, IQR/2—semi-interquartile range, Min–Max—range.

**Table 10:** Laboratory test results according to gender.

	Female		Male		Statistic	*p*-Value *
	Mdn	IQR	Mdn	IQR		
Serum albumin (g/dL)	2.4	1.2	3	1.3	0.738	0.456
CRP (mg/L)	21.9	56.7	16	57.9	−0.679	0.496
Total protein (g/L)	7	1.1	7	1.7	0.095	0.924
Haematocrit (%)	32.8	6.5	36.5	8.9	2.024	0.55

Mdn—median, IQR—interquartile range; * Brunner–Munzel test.

**Table 11:** Laboratory test results according to age.

	Spearman’s Rho	Degrees of Freedom	*p*-Value *
Serum albumin (g/dL)	−0.068	44	0.653
CRP (mg/L)	−0.055	44	0.718
Total protein (g/L)	0.025	44	0.871
Haematocrit (%)	0.171	43	0.261

* Spearman’s rank correlation coefficient.

**Table 12:** Laboratory test results with regard to the presence of stoma.

	Lack of Stoma		Presence of Stoma		Statistic	*p*-Value *
	Mdn	IQR	Mdn	IQR		
Serum albumin (g/dL)	2.8	1.6	3.0	1.2	0.153	0.895
CRP (mg/L)	17.9	51.8	23.3	99.7	0.936	0.36
Total protein (g/L)	7	1.3	7.4	1.3	1.595	0.125
Haematocrit (%)	33.7	6.2	33.2	9.9	−0.126	0.92

Mdn—median, IQR—interquartile range; * Brunner–Munzel test.

**Table 13:** Laboratory test results according to the type of SBS and indication for HPN.

Variable		Mdn	IQR	Stat.	*p*-Value *
	Indication for HPN				
Serum albumin	SBS	3.10	1.50	3.685	0.297
	Fistulas	2.40	0.75		
	Inefficient enteral nutrition	2.40	1.00		
	SBS and fistulas	3.20	0.93		
CRP	SBS	15.99	49.50	3.524	0.318
	Fistulas	17.93	6.10		
	Inefficient enteral nutrition	38.70	61.58		
	SBS and fistulas	39.64	44.55		
Total protein	SBS	7.10	1.15	12.544	0.006
	Fistulas	6.22	0.91		
	Inefficient enteral nutrition	6.80	0.70		
	SBS and fistulas	8.75	0.43		
Haematocrit	SBS	33.70	6.08	3.307	0.347
	Fistulas	30.30	8.23		
	Inefficient enteral nutrition	36.20	7.20		
	SBS and fistulas	36.50	8.10		
	Type of SBS **				
Serum albumin	No SBS	2.40	1.30	7.340	0.119
	SBS-J	2.75	0.53		
	SBS-JC ***	1.65	0.25		
	SBS-JIC	3.40	1.70		
	SBS-I	3.40	1.35		
CRP	No SBS	21.87	46.00	7.350	0.118
	SBS-J	35.87	91.71		
	SBS-JC ***	69.15	53.16		
	SBS-JIC	4.10	2.93		
	SBS-I	22.33	68.87		
Total protein	No SBS	6.40	1.20	7.350	0.118
	SBS-J	7.25	1.30		
	SBS-JC ***	5.55	1.15		
	SBS-JIC	7.30	2.70		
	SBS-I	7.31	0.73		
Haematocrit	No SBS	33.25	6.59	3.670	0.543
	SBS-J	32.00	7.00		
	SBS-JC ***	31.55	0.15		
	SBS-JIC	37.60	2.40		
	SBS-I	33.45	8.00		

Mdn—median, IQR—interquartile range; * Kruskal–Wallis rank test; ** SBS-J (short bowel syndrome with end jejunostomy), SBS-JC (short bowel syndrome with jejunocolic anastomosis), SBS-JIC (short bowel syndrome with jejunoileal anastomosis with an intact colon), SBS-I (short bowel syndrome with end ileostomy); *** SBS-JC values are not based on the median but represent the mean of two data points. Similarly, the interquartile range (IQR) for SBS-JC was calculated using these two values as the first (Q1) and third quartiles (Q3). Given the limited sample size, these values should be interpreted with caution.

**Table 14:** Composition of parenteral admixtures.

	N	M	SD	Mdn	IQR/2	Min	Max
Volume (mL)	45	2454.56	469.46	2600.00	375.00	1600.00	3200.00
Osmolarity (mosm/L)	45	858.64	133.11	835.40	78.10	565.20	1272.60
Energy (kcal)	45	1381.00	203.09	1398.00	94.00	466.00	1782.00
Non-protein energy (kcal)	45	1175.78	190.72	1198	66.5	266	1582
Amino acids (g)	45	50.93	7.21	50	3.5	30	62.5
Volume of fat emulsion (mL) *	45	134.44	46.25	100	25	50	300

M—mean, SD—standard deviation, Mdn—median, IQR/2—semi-interquartile range; * 20% emulsion.

**Table 15:** Composition of parenteral admixtures according to gender.

	Female		Male		Statistic	*p*-Value *
	Mdn	IQR	Mdn	IQR		
Volume (mL)	2150.0	498.0	2663.0	988.0	1.362	0.181
Osmolarity (mosm/L)	861	105	781	182	−1.574	0.118
Energy (kcal)	1398	176	1405	147	1.352	0.924
Non-protein energy (kcal)	1198	142	1198	96	2.024	0.55
Amino acids (g)	50.0	14.5	50.0	7.0	0.968	0.317
Volume of fat emulsion (mL)	100.0	50.0	125.0	87.5	0.442	0.665

Mdn—median, IQR—interquartile range. * The *p*-values were derived from the Brunner–Munzel test, utilising random permutation to assess the statistical significance of differences between two independent samples.

**Table 16:** Composition of parenteral formulas according to age.

	Spearman’s Rho	Degrees of Freedom	*p*-Value *
Volume (mL)	0.060	43	0.697
Osmolarity (Mosm/L)	−0.271	43	0.071
Energy (kcal)	−0.203	43	0.180
Non-protein energy (kcal)	−0.182	43	0.231

* Spearman’s rank correlation coefficient.

**Table 17:** Composition of parenteral formula in patients with and without stoma.

	Lack of Stoma		Presence of Stoma		Statistic	*p*-Value *
	Mdn	IQR	Mdn	IQR		
Volume (mL)	2150.0	545.0	2875.0	950.0	2.374	0.027
Osmolarity (mosm/L)	861.0	107.0	775.0	193.0	−1.513	0.134
Energy (kcal)	1398.0	196.0	1437.0	132.0	1.604	0.119
Non-protein energy (kcal)	1198.0	166.0	1215.0	96.5	1.258	0.211
Amino acids (g)	50.0	7.5	57.0	7.0	2.127	0.046
Volume of fat emulsion (mL)	50.0	7.5	57.0	7.0	2.127	0.046

Mdn—median, IQR—interquartile range. * The *p*-values were derived from the Brunner–Munzel test, utilising random permutation to assess the statistical significance of differences between two independent samples.

**Table 18:** Composition of parenteral formula according to the type of SBS and indication for HPN.

Variable		Mdn	IQR	Stat.	*p*-Value *
	Indication for HPN				
Volume (mL)	SBS	2400	742.50	5.450	0.142
	Fistulas	2650	431.25		
	Inefficient enteral nutrition	2150	450.00		
	SBS and fistulas	3000	263.75		
Osmolarity (Mosm/L)	SBS	862.1	186.95	4.200	0.240
	Fistulas	817.1	57.30		
	Inefficient enteral nutrition	861.4	56.75		
	SBS and fistulas	777.9	33.80		
Energy (kcal)	SBS	1362	214	4.790	0.188
	Fistulas	1437	80.75		
	Inefficient enteral nutrition	1407	38.75		
	SBS and fistulas	1479.5	52.75		
Non-protein energy (kcal)	SBS	1132	183	4.070	0.254
	Fistulas	1232	87		
	Inefficient enteral nutrition	1215	34		
	SBS and fistulas	1264.5	17.75		
Amino acids (g)	SBS	50	10.75	1.720	0.633
	Fistulas	53.5	7		
	Inefficient enteral nutrition	50	14.5		
	SBS and fistulas	53.5	8.38		
Volume of fat emulsion (mL)	SBS	100	50	4.79	0.188
	Fistulas	150	37.5		
	Inefficient enteral nutrition	150	50		
	SBS and fistulas	100	12.5		
	Type of SBS **				
Volume (mL)	No SBS	2600.00	500.00	5.880	0.209
	SBS-J	2622.50	812.50		
	SBS-JC ***	2100.00	500.00		
	SBS-JIC	2125.00	237.50		
	SBS-I	2650.00	937.50		
Osmolarity (Mosm/L)	No SBS	825.20	82.30	2.050	0.727
	SBS-J	829.55	199.78		
	SBS-JC ***	996.60	161.20		
	SBS-JIC	908.45	218.73		
	SBS-I	822.75	101.43		
Energy (kcal)	No SBS	1412.00	66.00	8.940	0.063
	SBS-J	1446.50	113.50		
	SBS-JC ***	1189.00	77.00		
	SBS-JIC	1266.00	113.75		
	SBS-I	1385.50	175.75		
Non-protein energy (kcal)	NO SBS	1232.00	66.00	7.980	0.092
	SBS-J	1231.50	92.50		
	SBS-JC ***	989.00	77.00		
	SBS-JIC	1066.00	100.00		
	SBS-I	1198.00	125.50		
Amino acids (g)	No SBS	50.00	7.00	4.880	0.300
	SBS-J	53.50	7.00		
	SBS-JC ***	50.00	0.00		
	SBS-JIC	46.25	10.63		
	SBS-I	50.00	14.50		
Volume of fat emulsion (mL)	No SBS	150.00	50.00	1.850	0.763
	SBS-J	150.00	50.00		
	SBS-JC ***	125.00	50.00		
	SBS-JIC	100.00	0.00		
	SBS-I	125.00	87.50		

* The *p*-values were obtained from the Kruskal–Wallis rank test, used to assess the statistical differences across multiple samples. ** SBS-J (short bowel syndrome with end jejunostomy), SBS-JC (short bowel syndrome with jejunocolic anastomosis), SBS-JIC (short bowel syndrome with jejunoileal anastomosis with an intact colon), SBS-I (short bowel syndrome with end ileostomy); *** SBS-JC values are not based on the median but represent the mean of two data points. Similarly, the interquartile range (IQR) for SBS-JC was calculated using these two values as the first (Q1) and third quartiles (Q3). Given the limited sample size, these values should be interpreted with caution.

**Table 19:** Complications of HPN according to gender.

	Female		Male		*p*-Value *
	N	%	N	%	
septic					0.767
yes	9	39.13	11	47.83	
no	14	60.87	12	52.17	
mechanical					0.414
yes	2	8.70	5	21.74	
no	21	91.30	18	78.26	
metabolic					0.489
yes	0	0.00	2	8.70	
no	23	100.00	21	91.30	

* Fisher’s exact test.

**Table 20:** Duration of HPN treatment (days) with regard to the cause of treatment discontinuation.

	N	Mean	Mdn	SD	IQR	Min	Max
Achieving intestinal autonomy	12	560	555	380.9	477.5	136	1419
Patient’s request	5	1383	572	1867.9	169	464	4722
Death	2	535	535	77.1	54.5	480	589
Lost to follow-up	1	NaN	NaN	NaN	NaN	NaN	NaN

M—mean, SD—standard deviation, Mdn—median, IQR/2—semi-interquartile range, CV—coefficient of variation, NaN—Not a Number (where some values for individual are absent or unknown).

## Data Availability

The data presented in this study are available on request from the corresponding author. The data are not publicly available due to ethical restrictions.
